# ASD-SAENet: A Sparse Autoencoder, and Deep-Neural Network Model for Detecting Autism Spectrum Disorder (ASD) Using fMRI Data

**DOI:** 10.3389/fncom.2021.654315

**Published:** 2021-04-08

**Authors:** Fahad Almuqhim, Fahad Saeed

**Affiliations:** Knight Foundation School of Computing and Information Sciences, Florida International University, Miami, FL, United States

**Keywords:** ASD, fMRI, autoencoder, sparse autoencoder, ABIDE, deep-learning, classification, diagnosis

## Abstract

Autism spectrum disorder (ASD) is a heterogenous neurodevelopmental disorder which is characterized by impaired communication, and limited social interactions. The shortcomings of current clinical approaches which are based exclusively on behavioral observation of symptomology, and poor understanding of the neurological mechanisms underlying ASD necessitates the identification of new biomarkers that can aid in study of brain development, and functioning, and can lead to accurate and early detection of ASD. In this paper, we developed a deep-learning model called *ASD-SAENet* for classifying patients with ASD from typical control subjects using fMRI data. We designed and implemented a sparse autoencoder (SAE) which results in optimized extraction of features that can be used for classification. These features are then fed into a deep neural network (DNN) which results in superior classification of fMRI brain scans more prone to ASD. Our proposed model is trained to optimize the classifier while improving extracted features based on both reconstructed data error and the classifier error. We evaluated our proposed deep-learning model using publicly available Autism Brain Imaging Data Exchange (ABIDE) dataset collected from 17 different research centers, and include more than 1,035 subjects. Our extensive experimentation demonstrate that *ASD-SAENet* exhibits comparable accuracy (70.8%), and superior specificity (79.1%) for the whole dataset as compared to other methods. Further, our experiments demonstrate superior results as compared to other state-of-the-art methods on 12 out of the 17 imaging centers exhibiting superior generalizability across different data acquisition sites and protocols. The implemented code is available on GitHub portal of our lab at: https://github.com/pcdslab/ASD-SAENet.

## 1. Introduction

More than 1.5 Million children (Baio et al., 2018) in the US are affected by heterogenous Autism Spectrum Disorder (ASD) which has wide range of symptoms or characteristics such as limited communication (including verbal and non-verbal), limited social interaction, and may exhibit repeated or limited interests or activities (American Psychiatric Association, 2013). Individuals with ASD have numerous challenges in daily life, and often develop comorbidities such as depression, anxiety disorder, or ADHD which may further complicate the diagnostic processes especially for young children (Mizuno et al., 2019). Although some symptoms are generally recognizable between 1 and 2 years of age; numerous children are not formally diagnosed with ASD diagnosis until they are much older (Stevens et al., 2016).

To date, the diagnostic process for individuals with ASD is based purely on behavioral descriptions of symptomology (DSM-5/ICD-10) (Nickel and Huang-Storms, 2017) from informants observing children with the disorder across different settings (e.g., home, school). Early cognitive, language, and social interventions for children (under 24 months old) with ASD has shown to be especially effective (Bradshaw et al., 2015), and a delayed diagnosis can have more disastrous effects in the life of the child. Help in terms of assisted learning or speech therapies is often available to these children (especially in low-income demographics), only *after* a diagnosis has been administered (Boat and Wu, 2015), making an early diagnosis even more urgent. ASD is associated with altered brain development in the early childhood but there are no reliable biomarkers that can be used for diagnosis (Lord et al., 2018). Collectively, our evolving understanding of the shared and distinct behavioral features that characterize ASD highlights the need for further inquiry into mechanisms behind ASD brain development, and functioning. The shortcomings of current clinical approaches (National Collaborating Centre for Mental Health, 2009), and the poor understanding of the neurological mechanisms underlying ASD necessitates the identification of new biomarkers and computational techniques that can aid clinicians, and neuroscientists alike to understand the distinct way ASD brain works as compared to a typical brain.

In the recent decade, advances in neuroimaging technologies are providing a critical step, and has made it possible to measure that functional and structural changes associated with ASD of the brain. To this end, functional Magnetic Resonance Imaging (fMRI) has been shown to demonstrate functional connectivity in brains with ASD compared to typical control brains (Eslami and Saeed, 2019). These functional images intrinsically has the data that can distinguish between ASD, and healthy controls but these subtle changes makes it impossible to distinguish distinctive biomarker patterns using conventional radiological readings, or even conventional computational, and statistical methods (Eslami and Saeed, 2019). Machine learning algorithms have been successful in identifying biomarkers from brain imaging structural Magnetic Resonance Imaging (sMRI) and functional Magnetic Resonance Imaging (fMRI) datasets for diagnosing brain disorders, such as ASD, ADHD, and Alzheimer's (Deshpande et al., 2015; Sarraf and Tofighi, 2016; Dvornek et al., 2017; Eslami and Saeed, 2018, 2019; El-Gazzar et al., 2019a; Yao and Lu, 2019).

In the recent decade, advances in neuroimaging technologies are providing a critical step, and has made it possible to measure the functional and structural changes associated with ASD (Just et al., 2007). Functional magnetic resonance imaging (fMRI) is commonly used to detect biomarker patterns for brain disorders (Just et al., 2007; Dichter, 2012; Botvinik-Nezer et al., 2020), and has gained extensive attention for ASD biomarker discovery, and classification (Iidaka, 2015; Plitt et al., 2015; Li et al., 2018; Xiao et al., 2018; El-Gazzar et al., 2019b; Wang et al., 2019). The fMRI data is shown to provide significant insights, and can demonstrate both the hypo- and hyper-connectivity in the ASD brain development (Di Martino et al., 2014; Lau et al., 2019), and can be used to study different origination theories related to ASD (Just et al., 2007). In fMRI studies, functional connectivity is based on the correlation of the activation time series in pairs of brain areas and are studied for both ASD and healthy brains. However, it is not possible to detect subtle biomarker patterns using conventional computational and statistical methods (Eslami and Saeed, 2019; Haweel et al., 2020; Nogay and Adeli, 2020). Machine learning algorithms have been successful in identifying biomarkers from functional Magnetic Resonance Imaging (fMRI) datasets for biomarker discovery, and classification of various brain disorders (Deshpande et al., 2015; Sarraf and Tofighi, 2016; Dvornek et al., 2017; Eslami and Saeed, 2018, 2019; El-Gazzar et al., 2019a; Yao and Lu, 2019). Effective modeling of ASD brain connectivity using fMRI data may lead to biomarker detection, and consequently better understanding of the brain neural activity associated with ASD.

In this study, we focus on designing and developing a machine-learning model that can distinguish and classify fMRI data from ASD subjects, and from typical control (TC). We focus on designing a deep learning algorithm that can extract, and distinguish between the functional features associated with ASD fMRI brain scans as compared to healthy typical controls. To this end, we have designed and implemented a deep-learning model, called *ASD-SAENet*, consisting of a sparse autoencoder (SAE) which lowers the dimensionality of our input features. The sparsity of SAE helps in extracting the features from high-dimensional imaging data while ensuring that limited sample size does not lead to overfitting (Ng, 2011). Extraction of feature(s) is then followed by a deep-neural network with 2-hidden layers, and softmax layer at the output. Our extensive experimentation using ABIDE-I datasets show that ASD-SAENet achieves an average accuracy of 70.8% improving upon our earlier state-of-the-art work- (Eslami et al., 2019), as well as other methods (Heinsfeld et al., 2018). In this study, we further demonstrate that ASD-SAENet model outperforms other methods (Heinsfeld et al., 2018; Eslami et al., 2019) in more than 12 of the 17 individual imaging sites showing superior generalizability across different data acquisition sites, protocols, and processes.

The structure of the paper is as follows: In section 2, we discuss the related work, and the associated state-of-the-art tools. In section 3, we propose the design of the machine-learning algorithm, the feature extraction, and the classification processes for the proposed ASD-SAENet method. Experimental results, datasets, and comparison against state-of-the-art methods are discussed in section 4. Section 5 illustrates the discussion, conclusions, and future work.

## 2. Related Work

Detecting, and finding biomarkers from imaging datasets such as fMRI has attracted significant attention in recent years. One of the key reasons of this increased attention, apart from the significance of finding quantitative biomarkers for ASD that can lead to new neuroscientific knowledge discovery, is the availability of publicly accessible Autism Brain Imaging Data Exchange (ABIDE) datasets collected from 17 different sites (Craddock et al., 2013) resulting in numerous studies (Abraham et al., 2017; Fredo et al., 2018; Khosla et al., 2018; El-Gazzar et al., 2019a; Parikh et al., 2019; Sherkatghanad et al., 2019).

Several studies used a *subset* of ABIDE dataset include El-Gazzar et al. (2019b) developed a 3D convolutional neural network and a 3D convolutional LSTM to classify ASD subjects from heathy subjects using fMRI data. The study was evaluated on a subset of ABIDE dataset containing 184 subjects collected from NYU and UM sites achieving 77% accuracy. In another work, Guo et al. (2017) proposed a deep neural network that has several stacked sparse autoencoders to lower dimensional features and a deep neural network (DNN) model to classify ASD patients from TC patients. The model was trained and tested on the dataset of UM site from ABIDE dataset, achieving 86.36% accuracy. Using the whole dataset from ABIDE, Brown et al. (2018) developed a model based on BrainNetCNN with an element-wise layer attached as the first step, and a data-driven structural priors. This model was evaluated on 1,013 subjects where 539 were heathy and 474 were ASD subjects and achieved an accuracy of 68.7%. Machine learning methods such as Support Vector Machine (SVM), and Random Forests have also been used to classify ASD subjects. Kazeminejad and Sotero (2019) obtained an accuracy of 95% by developing a feature selection pipeline based on graph theoretical metrics, and SVM method for classification. However, these results (Kazeminejad and Sotero, 2019) were based on a *subset* of ABIDE data set with subjects older than 30 years, and its generalizability is unknown for subjects that are children.

Deep learning techniques such as Deep learning network (DNN), Autoencoders, and Convolutional Neural Network (CNN) have gained an extensive attention for ASD biomarker detection, and classification studies (Khosla et al., 2018; Li et al., 2018; Wang et al., 2019; Yao and Lu, 2019). Heinsfeld et al. (2018) used a deep learning method which consists of two stacked denoising autoencoders, and a multi-layer classifier which achieved an average of 70% accuracy using the full ABIDE dataset. They also executed the model for each site, and they achieved an average accuracy of 52%. Recently, we (Eslami et al., 2019) proposed a deep-learning model called *ASD-DiagNet* which is the current state-of-the-art method in the field used by multiple studies (Mostafa et al., 2019a,b; Bilgen et al., 2020; Niu et al., 2020). ASD-DiagNet consists of an autoencoder for lowering the features dimensionality, and a single layer perceptron for classification decision. The method was trained with an expanded training data using data augmentation technique which then achieved 70.3% accuracy using the complete ABIDE dataset. In Eslami et al. (2019), accuracy for each of the 17 sites from ABIDE dataset resulted in average accuracy of 63.2%. However, we also reported that *ASD-DiagNet* exhibited 82% as the maximum accuracy for some of the sites which was 28% higher than any other method. However, clearly higher accuracy depicted by *ASD-DiagNet* still requires that the accuracy is generalizable across different sites with different MRI machines, and various data acquisition protocols, and pre-processing workflows. In this paper, we demonstrate that the proposed ASD-SAENet model exhibits comparable average accuracy relative to other methods but results in higher accuracy for 12 out of the 17 data acquisition centers. We will use these two studies (Heinsfeld et al., 2018; Eslami et al., 2019) to compare, and evaluate our proposed model.

## 3. Materials and Methods

### 3.1. Functional Magnetic Resonance Imaging and ABIDE Dataset

Functional Magnetic Resonance Imaging (fMRI) is a non-invasive brain imaging technique that allows capturing brain activity over time. The data of fMRI is represented by measuring the blood-oxygen-level-dependent (BOLD) volume of each small cubic called voxel at a given time point. Therefore, the data consists of a time series of each voxel representing its activity over time. For brain disorders, resting state fMRI (rs-fMRI) is commonly used which is scanning the brain image while the subject is resting. In this paper, we used the ABIDE-I dataset that is provided by the ABIDE initiative. This dataset consists 1,035 rs-fMRI data with 505 ASD subject, and 530 healthy control subjects that are collected from 17 different sites. The dataset was preprocessed and downloaded from (http://preprocessed-connectomes-project.org/abide/). We used the preprocessed data using the Configurable Pipeline for the Analysis of Connectomes C-PAC pipeline (Craddock et al., 2013) which is parcellated into 200 region of interests (ROIs) using Craddock 200 (CC200) functional parcellation (Craddock et al., 2012). For each region, the average voxels' BOLDs is calculated. The preprocessing steps also include skull-striping, slice time correction, motion correction, and nuisance signal regression. Each site used different parameters, and scanners for brain imaging, such as repetition time (TR), echo time (TE), and flip angle degree. Table 1 shows the parameters of each site.

**Table 1:** The scanning parameters of ABIDE dataset for each site show the different in MRI Scanner, TR (Repetition Time), TE (Echo Time), Flip Angle, and Age which may result in difference in data acquisition as well as the pre- and post-processing of fMRI data.

**Site**	**MRI scanner**	**TR (ms)**	**TE (ms)**	**Flip angle (degree)**	**Age (year)**
Caltech	SIEMENS	2,000	30	75	17–56.2
CMU	SIEMENS	2,000	30	73	19–40
KKI	PHILLIPS	2,500	30	75	8–12.8
Leuven	PHILLIPS	1,656	33	90	12.1–32
MaxMun	SIEMENS	3,000	30	80	7–58
NYU	SIEMENS	2,000	15	90	6.5–39.1
OHSU	SIEMENS	2,500	30	90	8–15.2
OLIN	SIEMENS	1,500	27	60	10–24
PITT	SIEMENS	1,500	25	70	9.3–35.2
SBL	PHILLIPS	2,200	30	80	20–64
SDSU	GE	2,000	30	90	8.7–17.2
Stanford	GE	2,000	30	80	7.5–12.9
Trinity	PHILLIPS	2,000	28	90	12–25.9
UCLA	SIEMENS	3,000	28	90	8.4–17.9
UM	GE	2,000	30	90	8.2–28.8
USM	SIEMENS	2,000	28	90	8.8–50.2
Yale	SIEMENS	2,000	25	60	7–17.8

*These differences may lead the machine-learning models learn site-specific variations leading many machine-learning models give better average accuracy (for whole ABIDE data set) than the site-specific accuracy*.

### 3.2. Feature Extraction

Craddock 200 (CC200) (Craddock et al., 2012) atlas divides the brain into 200 regions. Time series of each regions was extracted. Pearson's correlation coefficient is used to calculate the functional correlations of the ROIs. The following equation was used to obtain the correlation between two different time series data of each region *i*, and *j* of length *T*.

(1) $$\begin{array}{l} p_{i,j}=\frac{\sum_{t=1}^{T}\left(i_{t}-ī\right)\left(j_{t}-\bar{j}\right)}{\sqrt{\sum_{t=1}^{T}{\left(i_{t}-ī\right)}^{2}}\sqrt{\sum_{t=1}^{T}{\left(j_{t}-\bar{j}\right)}^{2}}} \end{array}$$

where $\bar{i}$, and $\bar{j}$ are the mean of the time series *i* and *j* respectively. A matrix *C*_n*n_ is obtained after computing all pair-wise correlations. Since we used CC200 atlas which divides the brain into n = 200 regions, it generates a matrix of 200 × 200. Due to the symmetry of the matrix with regard to the diagonal, we only consider the right upper triangle of the matrix, and flatten it to one-dimensional vector as features. These pairs result in (*n*)(*n* − 1)/2 = 19, 900 values for each vector. In order to reduce the dimensionality of the input, we adopted the same technique as in Eslami et al. (2019) and only considered 1/4 largest and 1/4 smallest of the average correlations, resulting in a vector of 9,950 values as the input for each subject.

### 3.3. Model Architecture: Feature Selection and Classification

In order to reduce the dimensionality of the input, we developed an autoencoder model. Autoencoder (AE) neural network is form of unsupervised learning that uses a feed-forward neural network with encoding, and decoding architecture. It is trained to get an input *x* and then reconstruct *x*′ to be as similar to the input *x* as possible. There are several types of autoencoders, such as sparse autoencoder (Ng, 2011), a stacked autoencoder (Vincent et al., 2010), and denoising autoencoder (Vincent et al., 2008). Autoencoders can fail to reconstruct the raw data since it might fall into copying task specially when there is a large data space. The lower-out put dimensions of a sparse autoencoder can force the autoencoder to reconstruct the raw data from useful features instead of copying it (Goodfellow et al., 2016b). For this study, we choose a sparse autoencoder which will be used to extract useful patterns with lower dimensionality. These feature vectors are then fed to our deep neural network model which consists of two hidden layers, and a softmax output layer.

**Figure 1:**
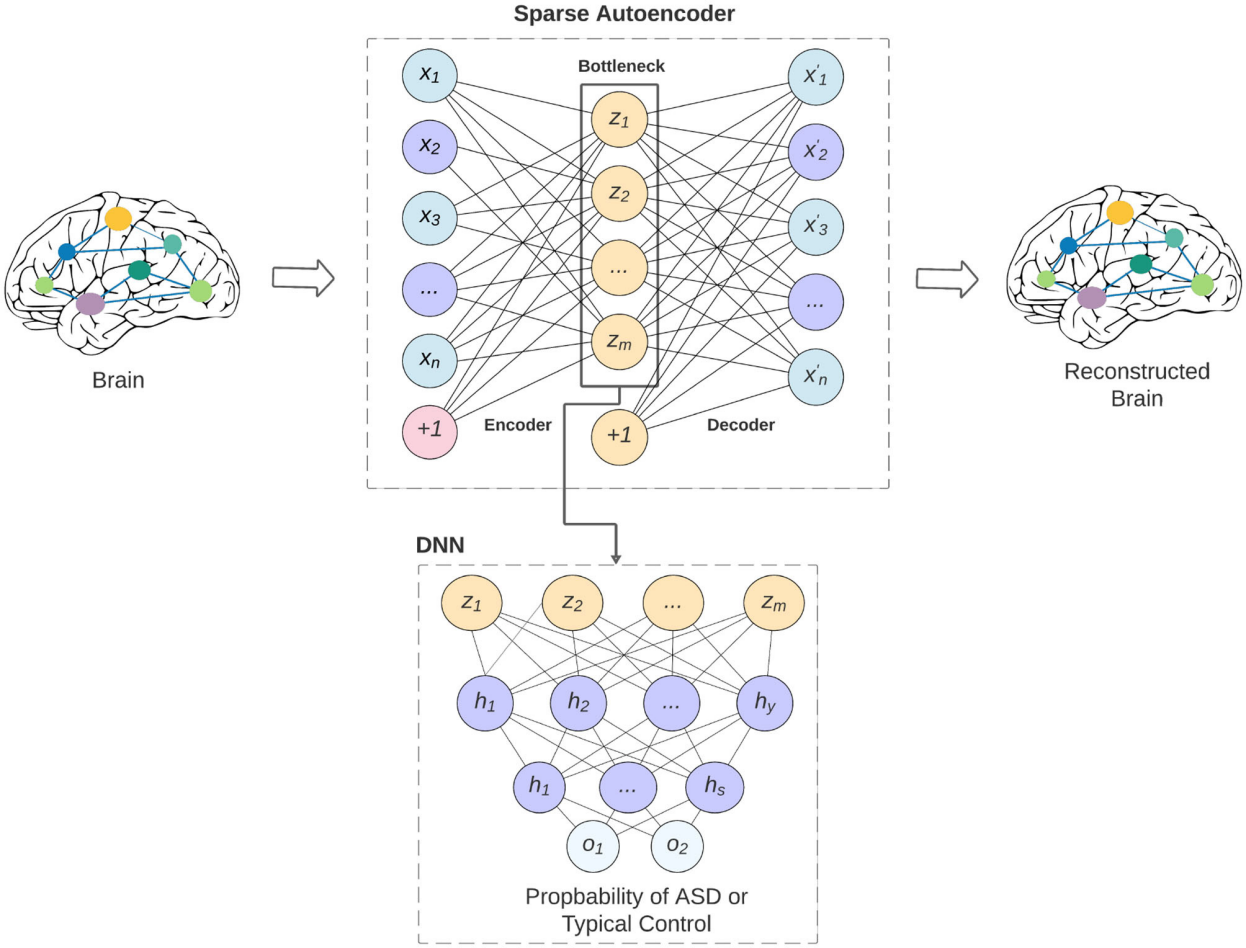
An overview of our proposed model and how the sparse autoencoder is used as feature selection to the deep neural network. The limitation of the feature lead to better generalizability of the model across various data-acquisition sites, and may lead to better interpretability of the models.

The overview of our model is shown in Figure 1. The bottleneck of the sparse autoencoder is used as input vector to the deep neural network. In the figure, neurons labeled as (+1) are the bias units added to the feed-forward neural network through the cost function. This step will force the AE to better reconstruct the input *x* without falling into overfitting. Our proposed sparse autoencoder's (SAE) cost function consists of three parts that are discussed below.

Given a dataset of *N* training samples (*x*_1_, *x*_2_, …*x*_*n*_), where *x*_*i*_ represents the *i*^*th*^ input. The developed SAE is trained to reconstruct the input *x*_*i*_ with the function *h*_*W,b*_(*x*_*i*_) to be as close to *x*_*i*_ as possible. The three parts of the cost function are mean squared error, weight decay, and sparsity term. The first two parts of the cost function, the mean squared error of all *N* training samples, and the weight decay can be defined as follows:

(2) $$\begin{array}{l} J_{sparse}\left(W,b\right)=\frac{1}{N}\overset{N}{\underset{i=1}{\sum }}\frac{1}{2}\left\|h_{W,b}\left(x^{i}\right)-x^{i}\right\| \end{array}$$

(3) $$\begin{array}{l} +\frac{\lambda }{2}\overset{n_{l}-1}{\underset{l=1}{\sum }}\overset{s_{l}}{\underset{i=1}{\sum }}\overset{s_{l+1}}{\underset{j=1}{\sum }}{\left(W_{ji}^{l}\right)}^{2} \end{array}$$

The Equation (3) defines the weight decay, which helps to avoid overfitting. A small value of λ may lead to overfitting, while a large value of λ may lead to underfitting. Thus, we performed several empirical experiments to select lambda to achieve the best fit of this term.

The third part of the cost function is the sparsity term, which is used to apply activations to the hidden layer of the autoencoder model to prevent overfitting. It can limit the number of regions that are considered in the hidden layer. The following equation defined the average activated value of the hidden layer were *a* denotes to the activation function which is rectifier (ReLU):

(4) $${\hat{p}}_{j}=\frac{1}{N}\sum_{i=1}^{N}(a_{j}^{2}(x^{i})$$

Now, the sparsity term is calculated to make ${\hat{p}}_{j}$ as close to *p* as possible, where *p* is the sparsity parameter. The benefit of this parameter is to deviate ${\hat{p}}_{j}$ from *p* which will result to activate and deactivate neurons on the hidden layer. This term is defined using Kullback-Leibler divergence as follows:

(5) $$\sum_{j=1}^{s_{l}}KL(p\|{\hat{p}}_{j})=\sum_{j=1}^{s_{l}}\left[plog\frac{p}{{\hat{p}}_{j}}+(1-p)log\frac{1-p}{1-{\hat{p}}_{j}}\right]$$

Finally, the cost function of our SAE model after adding all the three parts is defined as follows:

(6) $$\begin{array}{l} J_{sparse}(W,b)=\frac{1}{N}\sum_{i=1}^{N}\frac{1}{2}\|h_{W,b}(x^{i})-x^{i}\|+\frac{\text{λ}}{2}\sum_{l=1}^{n_{l}-1}\sum_{i=1}^{s_{l}}\sum_{j=1}^{s_{l+1}}{\left(W_{ji}^{l}\right)}^{2}\\ \;+\beta \sum_{j=1}^{s_{l}}KL(p\|{\hat{p}}_{j}) \end{array}$$

where *β* is the sparse penalty term.

The SAE is used to reduce the dimensional representation of the input where the size of the input is 9,500 features. The bottleneck of the SAE provides useful features that can be used as inputs for our deep neural network classifier. The size of the bottleneck is 4,975 hidden units. The classifier consists of two hidden layers, and an output layer where the units sizes are 4,975, 2,487, 500, and 2, respectively. The output layer is a softmax regression (Goodfellow et al., 2016a) which represents the probability of each class. To avoid overfitting, we used dropout between the fully connected neural networks. Then we take the maximum probability between the two classes as the final decision of the classifier. We used Cross Entropy for calculating the cost function of the classifier, and added a weight decay term.

**Figure 2:**
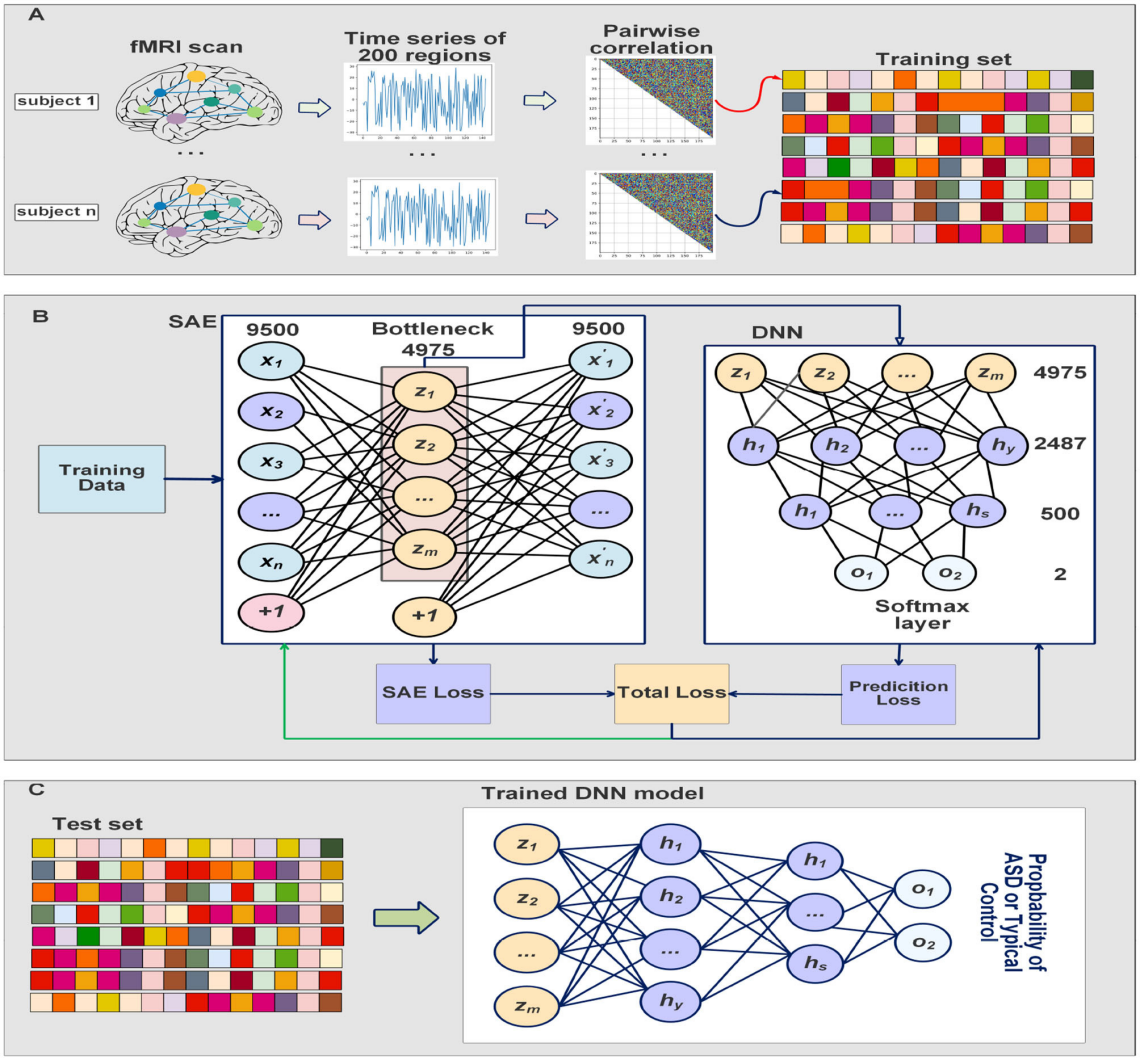
**(A)** Shows the preprocessing steps which include extract time series from fMRI scans, calculate the Pearson's correlations, and then 1/4 smallest and largest average pairwise correlations were selected for feature vectors. **(B)** Explain how our model is trained at the same time to improve feature selection while obtaining optimal classification model. The DNN classifier input is the bottleneck of the SAE. **(C)** Shows the testing process where the input subject is fed into the trained SAE, and then the DNN will take the bottleneck to make the classification using softmax layer.

The SAE is trained to minimize its cost function described above, and the deep neural network classifier is trained by taking the bottleneck of the SAE as inputs. The SAE and the classifier were trained simultaneously which results in feature extraction which improves while optimizing the classifier's decision. The training process is completed in 30 iterations and the batch size is 8. The sparsity parameter *p*, the weight decay λ, and the sparse penalty term *β* were chosen to be 0.05, 0.0001, and 2, respectively. We fine-tuned the deep neural network classifier on the last 10 iterations to adjust the parameters of the classifier, and minimize the cost function of the softmax while the parameters of SAE are frozen. Adam optimizer (Kingma and Ba, 2014) is used to update the parameters based on the computed gradients. Our ASD-SAENet model can be seen in Figure 2. All the experiments reported in this paper were performed using a Linux server with Ubuntu OS. The server has a processor of Intel Xeon E5-2690 v3 at 2.60 GHz. The total RAM is 54 GBs. The server also contains an NVIDIA Tesla K80 running CUDA version 10.2 and PyTorch library to perform our deep learning model.

### 3.4. Model Validation

Due to the limitation of the sample data, our model was evaluated using k-fold cross validation technique in which the dataset is randomly split into k equal sized samples, and one of these is used for getting the classification performance. This process is repeated k times to ensure that the model is not overfitted (Moore, 2001).

## 4. Experiments and Results

In our experiments, ASD-SAENet was evaluated in two different scenarios. First, the whole dataset containing 1,035 subjects were used to evaluate our model, and then we tested the model on each site separately. Evaluating each site separately demonstrates how our model performs on small datasets, and how it generalizes across different data acquisition sites and MRI machines. Due to the limitation of the sample data, our model was evaluated using k-fold cross validation technique in which the dataset is randomly split into k equal sized samples, and one of these is used for getting the classification performance. This process is repeated k times to ensure that the model is not overfitted (Moore, 2001). The details of each strategy are explained in the following subsections.

### 4.1. Average Accuracy for the ABIDE Dataset

In this experiment, we chose k as 10 to perform 10-fold cross validation using the whole dataset. We compare our proposed model with ASD-DiagNet model (Eslami et al., 2019), and the method proposed by Heinsfeld et al. (2018). Table 2 shows the comparison of accuracy, sensitivity, specificity, and the running time with these state-of-the-art tools. The result shows that ASD-SAENet achieves 70.8% which is comparable to the average accuracy of these methods.

**Table 2:** This table shows the comparison between ASD-SAENet and the state-of-the-art methods using the whole ABIDE dataset.

**Method**	**Accuracy (%)**	**Sensitivity (%)**	**Specificity (%)**	**Running time**
ASD-SAENet	**70.8**	62.2	**79.1**	52.74 min
ASD-DiagNet (Eslami et al., 2019)	70.3	68.3	72.2	41.14 min
Heinsfeld et al., 2018	70	**74**	63	7 h

*The bold values indicate the best accuracy, sensitivity, and specificity for the three models*.

### 4.2. Accuracy for Each Data Acquisition Site

In these experiments, we performed a 5-fold cross validation for each site because of the limitation of the size of the data. Table 3 shows accuracy, sensitivity, and specificity of ASD-SAENet for each site, and Table 4 compares the accuracy with other approaches. These results demonstrate that our proposed model outperforms other state-of-the-art methods, and exhibits better accuracy for 12 out of the 17 sites. The average accuracy achieved by ASD-SAENet model was 64.42% which is comparable to other methods as well. The fact that ASD-SAENet model exhibits better accuracy for more number of sites as compared to both state-of-the-art methods shows the robustness and generalizability of our proposed model.

**Table 3:** This table shows accuracy, sensitivity, and specificity using our proposed ASD-SAENet model for each imaging site of ABIDE dataset.

**Site**	**Accuracy (%)**	**Sensitivity (%)**	**Specificity (%)**
Caltech	56.7	68.3	45
CMU	70.6	93.3	46.6
KKI	72.6	60	82
Leuven	64.6	50.6	77.1
MaxMun	47.5	49	54
NYU	72	67.9	75
OHSU	72	50	90.3
OLIN	66.6	81.6	46.6
PITT	**73.1**	78.6	64.6
SBL	56.6	60	53.3
SDSU	64.2	53.3	65.9
Stanford	53.2	36.6	70
Trinity	57.5	48	64
UCLA	68.3	72.3	64.1
UM	67.8	79	58.3
USM	70	63.5	71.5
Yale	66	69.3	64
Average	64.6	63.6	64.2

*The bold value indicates the best accuracy among all the sites*.

**Table 4:** This table shows the comparison between ASD-SAENet and state-of-the-art methods ASD-DiagNet (Eslami et al., 2019), and Heinsfeld et al. (2018) for each site of ABIDE dataset.

**Site**	**Data size**		**Accuracy (%)**		
	**ASD**	**Typical control**	**ASD-SAENet**	**ASD-DiagNet (Eslami et al., 2019)**	**Heinsfeld et al., 2018**
Caltech	19	18	**56.7**	52.8	52.3
CMU	14	13	**70.6**	68.5	45.3
KKI	20	28	**72.6**	69.5	58.2
Leuven	29	34	**64.6**	61.3	51.8
MaxMun	24	28	47.5	48.6	**54.3**
NYU	75	100	**72**	68	64.5
OHSU	12	14	72	**82**	74
OLIN	19	15	**66.6**	65.1	44
PITT	29	27	**73.1**	67.8	59.8
SBL	15	15	**56.6**	51.6	46.6
SDSU	14	22	**64.2**	63	63.6
Stanford	19	20	53.2	**64.2**	48.5
Trinity	22	25	57.5	54.1	**61**
UCLA	54	44	68.3	**73.2**	57.7
UM	66	74	**67.8**	64.2	57.6
USM	46	25	**70**	68.2	62
Yale	28	28	**66**	63.8	57.6
Average			**64.6**	63.8	56.1

*The bold numbers indicate the best accuracy for the three models. As can be seen that ASD-SAENet exhibits better average-accuracy, as well as superior accuracy for 12 out of the 17 sites that are part of the ABIDE benchmark*.

## 5. Conclusions and Discussions

More than 1.5 Million children in the US are affected by heterogeneous Autism spectrum disorder (ASD) which has wide range of symptoms and characteristics such as limited communication (including verbal and non-verbal), limited social interaction, and may exhibit repeated or limited interests or activities. The diagnostic challenges using clinical techniques have resulted in significant interest in identifying a biomarkers, and consequently an objective test that correctly classifies children with and without the disorder earlier than the current timeline. However, before any such test can be administered at clinical level; sufficient understanding of the neurobiological underpinning of ASD is essential. In the recent decade, advances in neuroimaging technologies are providing a critical step, and has made it possible to measure that pathological changes associated with ASD brain. Imaging techniques such as structural MRI, and functional MRI (to detect the alterations in function, connectivity of the brain) can be used to detect the changes in the brain. The underlying fMRI data has the features that can be used to distinguish between ASD and healthy controls. However, the subtle changes in the ASD brain as compared to the healthy controls make it impossible to identify, and detect biomarkers using conventional computational or statistical methods. Advanced machine-learning solutions offer a systematic approach to developing automatic solutions for objective classification, and learn the subtle patterns in the data that might be specific to ASD brains.

In this paper, we have designed, and developed a deep-learning method, called *ASD-SAENet* for classifying brain scans that exhibit ASD from healthy controls scans. Our novel deep-learning model utilizes sparse autoencoders which are more open to interpretability, and may advance our understanding of the neurobiological underpinning of the ASD brain. The fMRI data used for training, and evaluating our deep-learning model is provided by ABIDE consortium, which has been collected from 17 different MRI data acquisition imaging centers. Our proposed model uses the Pearson's correlations of 200 regions of the brain as features which are fed into a sparse autoencoder to lower the dimensionality of the features. These features are then fed to the two-hidden layer deep-learning network with softmax function as output layer. Our proposed sparse autoencoder, and the deep-network is trained simultaneously for feature selection and improving classifier decision. Any further training to improve the classifier was done by executing more iterations with autoencoder kept at a constant state. Two major sets of experiments were performed for evaluation of our proposed model: First, we used the whole dataset and performed 10-fold cross-validation. We achieved 70.8% in 51 min which is significantly shorter than 6 hours required by other methods (Heinsfeld et al., 2018) while resulting in better accuracy. Second, we tested our method on each site using 5-fold cross-validation. The sparse autoencoder, coupled with limited amount of fMRI data that is available for ASD, demonstrates a computationally light-weight machine-learning module for ASD biomarker identification, and classification. Our extensive experimentation has shown that the proposed ASD-SAENet model gives higher accuracy for 12 out of the 17 centers that are part of ABIDE benchmark. Combined with the average accuracy of ASD-SAENet closer to the accuracy of state-of-the-art models (Heinsfeld et al., 2018; Eslami et al., 2019) conclusively shows that ASD-SAENet is a generalizable model, and is more robust to different data acquisition, various MRI machines, and (pre- and post-processing) protocols that are followed to acquire the fMRI data. Robustness of our ASD-SAENet model is a significant improvement to the variance observed in many existing state-of-the-art machine-learning models for ASD classification, and clearly suitable for further development for clinical usage in the future.

The variation in site-specific accuracies (shown in Tables 3, 4) can be explained by the variation in different data acquisition protocols (Power et al., 2012) involving different scanners, parameters, age range, as well as the post-data acquisition protocols that are followed by different groups Botvinik-Nezer et al. (2020). For example, in our results, the highest accuracy was on the PITT site dataset, which used Siemens scanner, repetition time of 1,500 ms, echo time of 25 ms, flip angle of 70 degrees, and an age range of 9.3–35.2. The lowest accuracy with almost similar data size was on MaxMun dataset which used same MRI scanner, different parameters (i.e., 3,000 for repetition time, 30 for echo time, and 80 for flip angle degree), and a huge gap of age range. Our results also show that different parameters and scanners can affect the quality of the data, and hence the performance of the deep-learning models. For example, most of the sites that achieved around 70% with our model were using Siemens scanner, repetition time is between 1,500 and 2,500, and age-range not highly variable. The results also demonstrate that there is a correlation between the echo time and the flip angle degree, and their sum between 95 and 105 gives the better performance for our deep-learning models. This empirical finding may show that Siemens scanner can work well when there is a correlation between echo time and flip angle degree. However, more studies and experiments are needed for confirmation, and how these data-acquisition parameters effect the features that are extracted by our deep-learning models.

Our extensive experimentation demonstrate that ASD-SAENet exhibits similar accuracy (70.8 vs. 70.3%) to our earlier proposed ASD-DiagNet (Eslami et al., 2019) model, superior specificity (79.1 vs. 72.2%) but slight decrease in sensitivity (62.2 vs. 68.3%). We attribute this slight decrease in sensitivity to the usage of sparse autoencoder in which only a small number of the hidden units are allowed to be active at the same time, and may miss some features. However, the strength of the model outweighs the small decrease in the sensitivity by exhibiting superior specificity. We also show that the number of true-negative rate is comparatively less than other state of the art methods; leading to classifiers that could be used in real-world i.e., since most of the population is not ASD, typical control people should be correctly identified as not having the condition. Additional advantages of the *ASD-SAENet* is the unique statistical features, and low computational cost which will help in identifying the feature importance estimates for our future studies. Absence of advance computational techniques such as interpretable deep-learning models that can process multimodal datasets such as sMRI and fMRI is a major technical hurdle in identifying ASD biomarkers. Investigation of these multimodal deep-learning models combined with the sparse autoencoder based classification strategy will allow us to device methods which will make the interpretation of the deep-learning models possible leading to better understanding of the neurobiological underpinning of the Autism Spectrum Disorder.

### 5.1. Limitations

This study has some limitations. First, although comparable to other published studies, the present study has a modest sample size for training and evaluation for our deep-learning model. Second, our model shows superior generalizability across multiple data acquisition sites but the features that might be most effective in classification cannot be determined due to non-interpretability of the deep-learning model. Third, there might be potential group differences due to head movement when using fMRI functional connectivity measures as input. The authors believe that such differences cannot be systematic due to variance in the subjects, scanning sites, and procedures that are site dependent. However, there is no way to demonstrate that the subtle changes picked up by deep-learning models are due to neurological difference, or due to head movements. We also believe that if a model achieves reliable classification accuracy (especially across different sites) despite such noise generated from different equipment and demographics shows promise for machine learning applications to ASD diagnosis and understanding. All of these limitations are at par with other machine-learning methods that have been proposed for biomarkers identification, and classification.

## Data Availability Statement

The datasets used in this study can be found in the ABIDE repository (http://preprocessed-connectomes-project.org/abide/).

## Author Contributions

FS conceived the study. FS and FA designed the machine-learning model and wrote and edited the manuscript. FA completed its implementations and reported results. All authors contributed to the article and approved the submitted version.

## Conflict of Interest

The authors declare that the research was conducted in the absence of any commercial or financial relationships that could be construed as a potential conflict of interest.
